# Primary Spontaneous Pneumothorax in Typhoid

**DOI:** 10.7759/cureus.11812

**Published:** 2020-11-30

**Authors:** Khalid L Khan, Shahid Ahmad, Mehr Nisa, Muhammad Hashim Peracha

**Affiliations:** 1 Family Medicine, Primary Health Care Corporation, Doha, QAT; 2 Ophthalmology, Primary Health Care Corporation, Doha, QAT

**Keywords:** pneumothorax, typhoid

## Abstract

We report a rare case of a 12-year-old girl who presented with a short history of diarrhea, vomiting and fever after traveling to Pakistan. During the course of initial investigations, her chest radiograph showed a primary spontaneous pneumothorax. There was no previous history of pulmonary disease. She was diagnosed as having *Salmonella* Typhi based on positive blood cultures. In the literature, spontaneous pneumothorax has been associated with typhoid fever as a complication of the disease in the pre-antibiotic era. However, a spontaneous pneumothorax associated with typhoid fever has never been reported to our knowledge in the post-antibiotic era.

## Introduction

Typhoid fever is a systemic disease caused by the bacteria *Salmonella* Typhi which spreads usually by ingestion of contaminated water or food [1,2]. The incidence of the disease has significantly reduced in the industrialized world, however, it is still common in the developing world [3]. The highest risk of the disease is in South Asian countries [1]. The disease is characterized by prolonged fever, headache, abdominal pain, rash and diarrhea or constipation. Rarely serious complications can occur which may lead to death [1,2]. Travelers to endemic areas are advised to take precautions including vaccination against the disease.

A 12-year-old girl presented with a short history of diarrhea, vomiting and fever after travelling to Pakistan. During the course of initial investigations, her chest radiograph showed a primary spontaneous pneumothorax (PSP). Primary spontaneous pneumothorax is defined as abnormal collection of air in the pleural space in the absence of underlying pulmonary disease or any injury, resulting in partial or complete collapse of the lungs [4]. There was no previous history of pulmonary disease in this case. She was diagnosed as having *Salmonella* Typhi based on positive blood cultures.

In the literature, pneumothorax has been associated with typhoid fever as a complication of the disease in the pre-antibiotic era. However spontaneous pneumothorax associated with typhoid fever has never been reported, to our knowledge, in the post-antibiotic era.

## Case presentation

A 12-year-old girl was seen in the family medicine clinic with a history of diarrhea, vomiting and fever for five days. There was no blood in the stool. She had recently travelled to Pakistan for a short holiday. She did not report any unusual physical activity. There was no history of previous primary spontaneous pneumothorax. Her examination was unremarkable with no signs of dehydration. Stool cultures showed no evidence of salmonella/shigella or ova and parasites. She had mild anemia of 10.7 gm/dl, mean corpuscular volume (MCV) 61.7 fl with a normal white cell count. She was known to have thalassemia minor. Initially she was treated symptomatically for traveller’s diarrhea but no antibiotics were prescribed.

**Figure 1:**
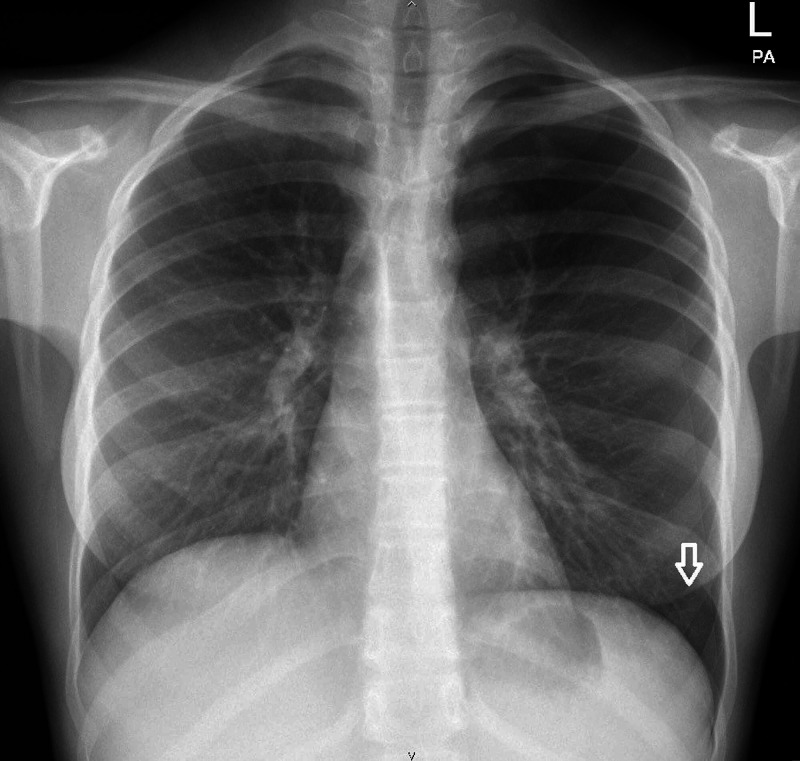
Chest X-ray showing left spontaneous pneumothorax.

**Figure 2:**
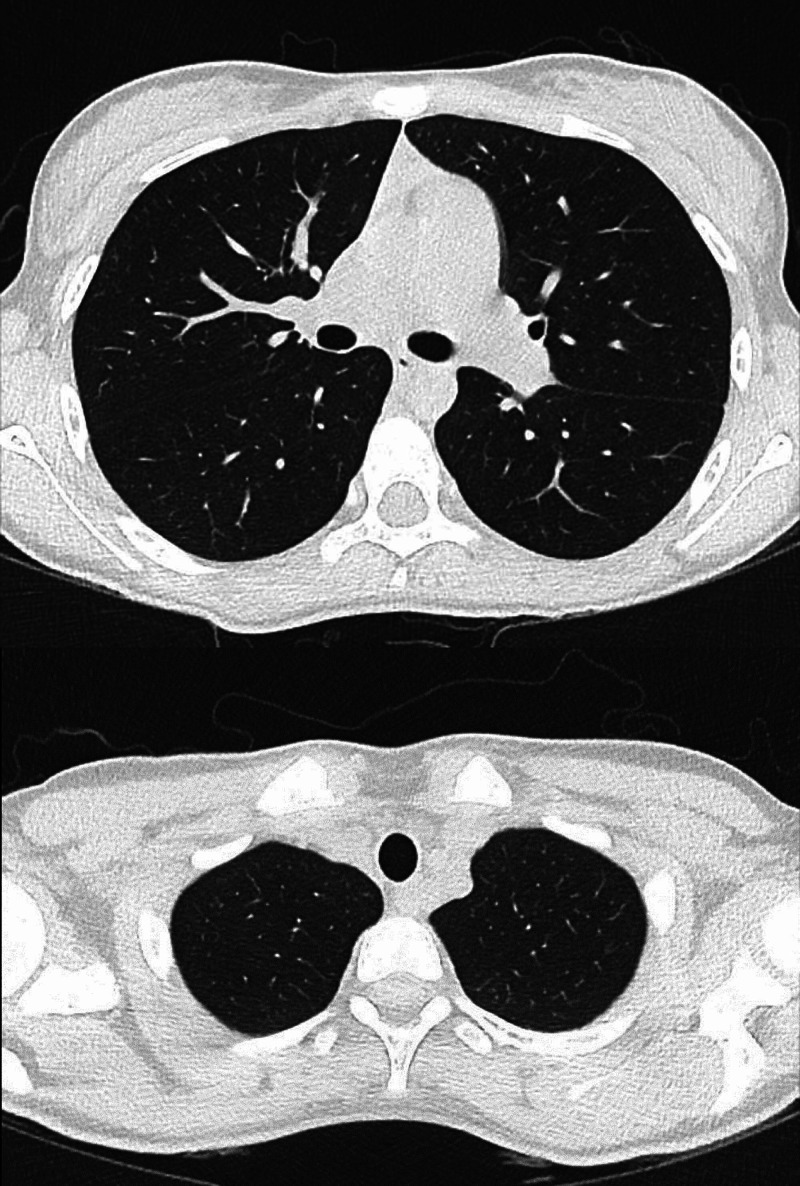
CT Thorax showing resolution of the pneumothorax and no evidence of blebs or bullae

She was reviewed the next day with no improvement in her symptoms and was noted to have a slight cough in the pediatric emergency department. There was no history of shortness of breath or chest pain. Auscultation revealed mild basal crackles. Chest radiograph and blood cultures were requested. Her chest x-ray revealed a left-sided large pneumothorax, with no evidence of any other radiographic abnormalities. (Figure 1). The patient was admitted to the hospital. On the next day, the blood cultures showed growth of *Salmonella* Typhi. A diagnosis of typhoid with an associated spontaneous pneumothorax was made. Her pneumothorax was managed conservatively, and a subsequent CT thorax after two weeks showed resolution of the pneumothorax. The CT scan showed no evidence of any parenchymal abnormalities, bullae, blebs or previous lung pathology (Figure 2). The patient’s *Salmonella* Typhi infection was treated with IV antibiotics (ceftriaxone 2 grams once daily) for two weeks which were started in hospital and later the course was completed in the community with complete recovery.

## Discussion

Typhoid fever is a disease caused by the bacterium *Salmonella enterica* serovar Typhi (*Salmonella* Typhi), which is transmitted through ingestion of food and water contaminated with faeces from patients with typhoid fever or carriers [5]. Global estimates indicate that 11-20 million individuals are infected with the disease, with 120,000-200,000 dying annually [6]. Extra-intestinal complication of typhoid disease is a known clinical phenomenon. A recent systematic review showed a higher pooled prevalence of typhoid fever complications from studies including children 27% (95% CI, 19%-35%) than from studies including adults 17% (95% CI, 9%-25%), although with a very high heterogeneity for both groups [7]. Among extra-intestinal complications, pulmonary complications are relatively common. In literature, bronchopneumonia as a pulmonary complication of typhoid has been widely reported [8-10]. However other complications such as acute respiratory distress syndrome (ARDS) [11], pulmonary haemorrhage [12], and pleural empyema [13] are less common. Pneumothorax was commonly seen in the pre-antibiotic era due to embolism or pyaemia [14] however rarely seen nowadays. Spontaneous pneumothorax with typhoid fever was, to our knowledge, reported for the first time by Harrington in 1886 after the isolation of *Salmonella* Typhi in 1884. In his paper published in the Journal of the American Medical Association (JAMA), he quotes Hoffman’s autopsies of typhoid patients at Basle revealing a case of pneumothorax [15]. 

The incidence of PSP in the pediatric population is around 3.4/100,000 with a male to female ratio ranging from 2:1 to 9:1 [16-17]. In pediatric studies, the peak age of incidence is between 14 and 17 years of age, mainly in late teenagers. The patients are typically tall and thin with a mean body mass index (BMI) of around 18 kg/m^2^, which is classified as underweight [18]. These tall and slim children may tend to have a higher transpulmonary pressure at the lung apex, and their rapid growth relative to pulmonary vasculature may result in ischemia and thus bleb formation at these regions [19], which can rupture leading to spontaneous pneumothorax. Our patient, however, was not tall height 155 cm i.e. 50th centile) but was slim with a weight of 43 kg and BMI of 16. 

Furthermore, apical bullae and subpleural blebs are found in the majority of PSP patients. In adult studies, subpleural blebs or bullae (usually at the apical portion of the upper lobe) are found in 76%-100% of patients during video-assisted thoracoscopic surgery (VATS) and in nearly all patients during thoracotomy [20]. However, no such findings were radiologically evident in our patient. It is however entirely plausible that she did have apical blebs which were not evident radiologically and due to her cough an acute increase in transpulmonary pressure led to the rupture of a bleb resulting in her pneumothorax. However, it has to be said that the exact pathogenesis of PSP until now still remains unclear.

## Conclusions

We postulate that given the paucity of features to support the known pathogenesis of PSP in our patient, her pneumothorax is likely a secondary complication of her typhoid fever, however the exact mechanism of this is unclear and needs to be studied further. 
